# Association between type 2 diabetes and chronic arsenic exposure in drinking water: A cross sectional study in Bangladesh

**DOI:** 10.1186/1476-069X-11-38

**Published:** 2012-06-07

**Authors:** Md Rafiqul Islam, Ismail Khan, Sheikh Md Nazmul Hassan, Mark McEvoy, Catherine D’Este, John Attia, Roseanne Peel, Munira Sultana, Shahnaz Akter, Abul Hasnat Milton

**Affiliations:** 1 Centre for Clinical Epidemiology & Biostatistics (CCEB), The University of Newcastle, Lot 1 Kookaburra Circuit, New Lambton Heights, NSW, 2305, Australia; 2 Department of Pharmacology, Dhaka Medical College, Dhaka, Bangladesh; 3 Department of Public Health, Atish Dipankar University of Science & Technology, Dhaka, Dhanmondi, Bangladesh; 4 Centre for Health and Development (CHAD), Gopalgonj, Bangladesh; 5 Institute of Child and Mother Health (ICMH), Dhaka, Bangladesh

## Abstract

**Background:**

Chronic exposure to high level of inorganic arsenic in drinking water has been associated with Type 2 Diabetes (T2D). Most research has been ecological in nature and has focused on high levels of arsenic exposure with few studies directly measuring arsenic levels in drinking water as an index of arsenic exposure. The effect of low to moderate levels of arsenic exposure on diabetes risk is largely unknown thus our study is adding further knowledge over previous works.

**Methods:**

This cross sectional study was conducted in 1004 consenting women and men from 1682 eligible participants yielding a participation rate of 60%. These participants are aged >30 years and were living in Bangladesh and had continuously consumed arsenic-contaminated drinking water for at least 6 months. T2D cases were diagnosed using glucometer following the new diagnostic criteria (Fasting Blood Glucose > 126 mg/dl) from the WHO guideline (WHO 2006), or a self-reported physician diagnosis of type 2 diabetes. Association between T2D and chronic arsenic exposure was estimated by multiple logistic regression with adjustment for age, sex, education, Body Mass Index (BMI) and family history of T2D.

**Results:**

A total of 1004 individuals participated in the study. The prevalence of T2D was 9% (95% CI 7-11%). After adjustment for diabetes risk factors, an increased risk of type 2 diabetes was observed for arsenic exposure over 50 μg/L with those in the highest category having almost double the risk of type 2 diabetes (OR=1.9 ; 95% CI 1.1-3.5). For most levels of arsenic exposure, the risk estimates are higher with longer exposure; a dose–response pattern was also observed.

**Conclusions:**

These findings suggest an association between chronic arsenic exposure through drinking water and T2D. Risks are generally higher with longer duration of arsenic exposure. The risk of T2D is highest among those who were exposed to the highest concentration of arsenic for more than 10 years.

## Background

Arsenic is a recognised carcinogen and toxicant [1,2]. It has a wide range of adverse health effects including cancer of the skin and internal organs, chronic bronchitis, hypertension, and skin lesions such as hyper pigmentation, and hyper keratoses. Chronic arsenic exposure has also been reported to be a potential risk factor for type 2 diabetes [3-6]. T2D is a metabolic disorder characterized by hyperglycaemia, insulin resistance in peripheral tissues, and altered insulin secretory capacity of pancreatic β cells [7,8]. Type 2 diabetes accounts for 90-95% of all cases of diabetes and is a major public health problem worldwide [9]. Established risks factors of T2D include older age, obesity, physical inactivity, family history, and genetic polymorphisms [10]. In addition, environmental toxicants, including arsenic, have been suggested to play an aetiologic role in developing diabetes [5,6,11], although this association is yet to be established.

Previous studies reported a possible association between chronic ingestion of arsenic in drinking water and T2D [5,6,12], although some of these studies are inconsistent, and the quality of the data has been questioned [9,11,13]. Recently, Chen et al., reported no association between arsenic exposure from drinking water and diabetes mellitus in a study conducted in Araihazar, an upazila (sub-district) of Narayangonj District, Dhaka, Bangladesh [14]. Experimental studies that explored the diabetogenic effects of inorganic arsenic also reported conflicting results [9,15-17]. Overall, the experimental and epidemiologic evidence is at present insufficient and inadequate to establish a causal association between arsenic exposure and T2D.

At least, 20% of the rural population of Bangladesh has been exposed to naturally occurring arsenic through drinking water [18]. From the public health context, it is therefore important to assess the role of arsenic in drinking water as a factor in the development of T2D in Bangladesh. Our cross sectional-analytical study was designed to examine the association between chronic arsenic exposure and T2D.

## Methods

### Overview

Bangladesh is divided into 64 districts, with each district divided into sub-districts named upazilas, and each upazila further divided into a number of unions. Each union consists of a number of villages, each with a number of tube wells from which the inhabitants draw their water for daily usage including drinking and cooking.

The study was conducted from January to July 2009. Ethics approval was obtained from the Bangladesh Medical Research Council (BMRC) and the Human Research Ethics Committee (HREC), The University of Newcastle, Australia.

### Study area and population

After careful review of the Bangladesh Arsenic Mitigation Water Supply Project’s (BAMWSP) national survey report, arsenic contaminated areas were selected from the Kandirpar, Gobindogonj, Uttarda and Modaffargonj union of the Laksam upazila, and the Jolmuttar union of the Monohargonj upazila of Comilla; the Sundorpur and Durgapur unions of the Kaliganj upazila of Jhenidah were chosen as low arsenic contaminated areas. A union having > 40% of the tube wells contaminated with arsenic >50 μg/L, of which 25% had arsenic more than 250 μg/L were considered as a high arsenic contaminated area for this study. A union with no tube wells containing arsenic at more than 50 μg/L was considered as a low arsenic contaminated area. These areas were chosen purposively to ensure the study population’s exposure to a wide range of arsenic that enabled us to study the effect of various levels of arsenic on T2D. Comilla and Jhenidah are 100 km south-east and 180 km south-west from Dhaka city, the capital of Bangladesh, respectively. One hundred households were randomly selected from each of the seven unions. Interviewers visited all selected households in each area. All individuals present during the household visit who fulfilled eligibility criteria were recruited in the study after their informed verbal consent was given. Eligibility criteria were as follows: aged >30 years, history of drinking water from any tube well for a least 6 months continuously, and resident in the study area for at least the preceding 6 months. Those aged less than 30 years or those with a chronic medical condition except T2D were deemed ineligible.

### Data collection procedure

Six trained field workers conducted a door to door survey to identify potential participants from the randomly selected households. Since the literacy rate is low in the selected sampling areas in Bangladesh, the study information sheet was read directly in front of potential participants, and the objectives and expectations of the study were comprehensively explained. Trained field workers then obtained informed verbal consent from the participants.

Interviewers collected information on socio-demographics, and drinking water use history by face to face interview using a structured questionnaire. The questionnaire was developed by the researchers and pretested in a neighbouring village similar to the study area. All participants underwent a full body examination by a well-trained physician under sunlight to investigate the presence of arsenical skin lesions i.e., melanosis, leucomelanosis and keratosis. Standing height and weight of each participant were measured with the subjects wearing light clothes and not wearing shoes. Weight was measured in kilograms using bathroom scales (Precision 500gm) that were calibrated daily and zeroed before each measurement. Height was measured in centimetres using a locally made wooden height board with a metal measurement tape attached. All measurements were made according to standard WHO procedures [19]. Fasting blood samples were collected from each participant. Interviewers confirmed the fasting status verbally before collecting the blood samples. All field workers were kept blind to the objectives of the study to reduce observer bias.

### Exposure measurement

A single well water measurement was used to characterize chronic arsenic exposure for each participant. We measured the arsenic content of each tubewell water. In case of participants who changed their arsenic contaminated tubewell following BAMWSP survey, we collected water samples from the previous tubewell as a measure for arsenic exposure. This however, only affected 20 participants. We also collected self-reported information on duration of drinking water from these selected tube wells. Cumulative arsenic (total arsenic) exposure was calculated by multiplying the arsenic content of the tube well with duration of use of the well. Drinking water samples were collected from the selected tube wells, transferred and analysed following standard procedure [20]. Water arsenic analyses were performed at a Water Quality Testing Laboratory in Dhaka using flow injection-hydride generation atomic absorption spectrometry (FIHG-AAS) following the standard procedure [20]. The process involves argon gas as carrier and electrical muffle heater in use to maintain temperature at 850° C. The hydride vapor generation involves 1% aqueous solution of Sodium borohydride and 5ml HCl while 15% aqueous solution of KI is used as reducing agent with one hour holding time before hydride generation. The quality control procedure to measure arsenic in this method involves several measures such as calibration checks, duplicate measurements and participation in inter laboratory comparison exercises. Minimum detection level of arsenic by this method is 3μg/L.

### Other variables

Information on socio-demographic characteristics such as age, sex, income, education and occupation, marital status, drinking water history, smoking history, height, weight, arsenical skin lesions, family history of T2D and hypertension were collected. BMI was also calculated using the standard method (weight in kg / height in meter^2^) [21].

### Outcome measurement

Participants were classified as having T2D according to the WHO standard diagnostic criteria: Fasting Blood Glucose > 126 mg/dl or a self-reported physician diagnosis of type 2 diabetes [22]. Fasting Blood samples were collected in the morning and analysed for blood glucose levels using an EZ Smart-168 Glucometer (Tyson Bioresearch, Taiwan). All T2D cases identified in this study were referred to the nearest health facilities for treatment and follow-up.

### Statistical analysis

Data were analysed using Stata statistical analysis software, version 10 (Stata, TX, USA, 2001). Frequency tables and summary statistics were obtained to check missing data, out of range values and to assess distributions of continuous variables, and logic checks were undertaken.

Multiple logistic regressions were done to determine the association between chronic arsenic exposure and T2D, adjusting for potential confounders. For these regression analyses, exposure was initially categorised as <50 μg/L and >50 μg/L, as 50μg/L is the maximum permissible limit of arsenic in drinking water for Bangladesh [23]; secondarily analyses were done using quartiles of arsenic levels. To assess the impact of duration of arsenic exposure (up to 10 years and more than 10 years), we categorized the arsenic concentration as <50 μg/L and >50 μg/L, and >50 μg/L arsenic concentration was sub categorized as 51–250 μg/L and >250 μg/L [24]. Initially univariate analyses such as chi-square tests and univariate logistic regression were undertaken to investigate the relationship between T2D and chronic arsenic exposure. Potential confounders such as participant’s age, sex, education (High school and above; primary class 1–5, no formal education), occupation (agriculture; day labour; housewives, other), marital status (currently married, widowed, divorced, never married, other), total number of household members, income (household’s total monthly income), roof type (straw, tin, concrete, other), smoking history (never, previous, current), body mass index ( <18.5 as malnourished and >18.5 as normal), and presence of arsenical skin lesions were included in the initial model. Only those variables found to be associated at *P*<0.25 level of significance in the initial model were included in the final multiple regression model. Additionally, we included BMI in the final regression model although it was not statistically significant at P <0.25 level of significance in the initial model as BMI is a known confounder for T2D. To estimate the likelihood ratio for model comparison adjusting for the clustering effect of tube well (household using the same well), we fitted a random effect logistic regression model using tube well id as the random effect variable. We used the random effect models to estimate likelihood ratio to compare between nested models while adjusting for clusters. After we came up with the final set of relevant covariates we refit a multiple logistic regression model where confidence intervals were estimated using the Huber/White robust ‘sandwich’ estimator of variance, which is based on the within cluster correlation observed in the data. Cuzick's nonparametric test for trend was used to test for trend across ordered groups.

## Results

A total of 1682 people were eligible to participate and 1004 actually agreed, yielding a participation rate of 60%. Information on baseline characteristics by their drinking water arsenic concentration is presented in Table 1. Participants’ mean age was 44.9 years (±13.2) and more females (68.4%) participated than males. Of the total, 69.1% participants received no formal education. The majority of the participants (67.2%) performed home duties. More than 98% of participants were married or previously married. Hindus were a minority (23.7%) among the study participants. Average total monthly income per household was 69.5 US$ (±66). Study participants were relatively lean with a mean BMI (kg/m^2^) of 20.3 (±4.5). Overall, 14.2% of the participants were current smokers, and mean duration of smoking was 22.6 years (±12.9). A total of 1004 drinking water samples were collected and analysed. All tubewell water contained arsenic above the minimum detection limit (3 microgram). An average of 5.5 (±2.2) persons was using each drinking water sample, with a range of 1 to 18 persons. Mean arsenic concentration of the tubewells were 159 μg/L (±198.5) ranging from 10 to 1401 μg/L. The overall prevalence of T2D among the study participants was 9% (95% CI 7%-11%), while 4.7% of the participants reported a family history of type 2 diabetes. Only 38% of the participants with T2D were already diagnosed, with the remaining 62% being diagnosed on blood tests during the study. Diabetic individuals were older and more likely to be current or ex-smokers (Table 2).

**Table 1:** Water arsenic concentration by participant’s characteristics (n=1004)^*^

Participant’s characteristics	Median arsenic Conc. in drinking water (μg/L)	Water arsenic concentration				No of subjects %, N
		1^st^ quartile	2^nd^ quartile	3^rd^ quartile	4^th^ quartile	
		(10–22 μg/L),%, N	(23–32 μg/L), %, N	(33–261 μg/L),%, N	(≥262 μg/L), %, N	
**Age**						
30–39	33	28.9 (117)	20.0 (81)	26.4 (107)	24.7 (100)	40.4 (405)
40–49	33	26.2 (69)	23.2 (61)	26.6 (70)	24.0 (63)	26.2 (263)
50–59	32	36.5 (57)	17.3 (27)	22.5 (35)	23.7 (37)	15.5 (156)
≥60	32	29.4 (53)	22.2 (40)	22.8 (41)	25.6 (46)	17.9 (180)
**Sex**						
Male	86	27.4 (87)	18.0 (57)	22.4 (71)	32.2 (102)	31.6 (317)
Female	32	30.4 (209)	22.1 (152)	26.5 (182)	21.0 (144)	68.4 (687)
**Education**						
No formal education	32	29.8 (207)	22.9 (159)	22.9 (159)	24.4 (169)	69.1 (694)
Primary	32.5	31.2 (58)	18.8 (35)	26.3 (49)	23.7 (44)	18.5 (186)
High School	117.5	25.0 (31)	12.1 (15)	36.3 (45)	26.6 (33)	12.4 (124)
**Occupation**						
Agriculture	109	28.0 (37)	13.7 (18)	26.5 (35)	31.8 (42)	13.1 (132)
Day labour	31	33.4 (17)	23.5 (12)	19.6 (10)	23.5 (12)	5.1 (51)
Housewife	32	30.5 (206)	22.7 (153)	26.1 (176)	20.7 (140)	67.2 (675)
Others	97	24.7 (36)	17.8 (26)	21.9 (32)	35.6 (52)	14.6 (146)
**Marital Status**						
Currently married	33	29.3 (253)	20.3 (175)	25.7 (221)	24.7 (213)	85.9 (862)
Widowed/Divorced	31	32.8 (43)	23.7 (31)	21.4 (28)	22.1 (29)	13.0 (131)
Never married	140	0.0 (0)	27.3 (3)	36.4 (4)	36.3 (4)	1.1 (11)
**Religion**						
Muslim	124	22.3 (171)	15.9 (122)	30.4 (233)	31.4 (240)	76.3 (766)
Hindu	83.5	26.5 (36)	18.4 (25)	22.8 (31)	32.3 (44)	14.2 (136)
**BMI**						
<18.5	109	25.1 (96)	16.2 (62)	27.8 (106)	30.9 (118)	38.0 (382)
18.5-25.0	31	33.5 (176)	22.6 (119)	22.0 (116)	21.9 (115)	52.4 (526)
≥25.0	32	25.0 (24)	29.2 (28)	32.3 (31)	13.5 (13)	9.6 (96)
**Smoking Status**						
Never smoked	32	31.5 (247)	22.6 (177)	24.2 (190)	21.7 (170)	82.1 (784)
Ex smoker	67	31.5 (11)	17.1 (6)	17.1 (6)	34.3 (12)	3.7 (35)
Current smoker	83.5	26.5 (36)	18.4 (25)	22.8 (31)	32.3 (44)	14.2 (136)
**Diabetes in the family**						
Yes	122	25.5 (12)	17.0 (8)	29.8 (14)	27.7 (13)	4.7 (47)
No	32	29.7 (284)	21.0 (201)	25.0 (239)	24.3 (233)	95.3 (957)

* Results are expressed as row percent, unless otherwise indicated.

**Table 2:** Distribution of characteristics of the study population by diabetes status (n=1004)

Characteristics	Diabetes (n = 89)	No Diabetes (n = 915)
Men (n, %)	41 (12.9)	276 (87.1)
Age (in years, mean ±sd)	47.3 ± 13.3	44.6 ± 13.2
Education (n, %)		
No formal education	55 (61.8)	639 (69.8)
Primary	19 (21.3)	167 (18.3)
>Primary	15 (16.9)	109 (11.9)
Occupation (n, %)		
Agriculture	19 (21.3)	113 (12.4)
Daily labourer	4 (4.5)	47 (5.1)
Housewife	47 (52.9)	628 (68.6)
Others	19 (21.3)	127 (13.9)
Marital status (n, %)		
Currently married	80 (89.9)	782 (85.5)
Widowed	8 (9.0)	119 (13.0)
Divorced	0 (0.0)	4 (0.4)
Never married	1 (1.1)	10 (1.1)
Religion		
Islam	77 (86.5)	689 (75.3)
Hindu	12 (13.5)	77 (24.7)
Total monthly household income (US$)^*^		
mean + SD	74 ± 77	69 ± 65.6
Median	51.5	51.5
Roof type^*^ (n, %)		
Tin	79 (88.8)	805 (88.0)
Others	10 (11.2)	109 (12.0)
Body Mass Index (BMI); mean + SD	19.8 + 3.4	20.3 + 4.6
Smoking status^*^ (n, %)		
Current smokers	17 (20.0)	119 (13.7)
Ex smokers	6 (7.1)	29 (3.3)
Never smoked	62 (72.9)	722 (83.0))
Duration of smoking (years)^*^; mean + SD		
Current smokers	22.5 ± 11.9	22.5 ± 13.2
Ex smokers	18.3 ± 10.8	19.3 ± 10.5
Family history of Type 2		
Diabetes (n, %)		
Yes	1 (1.1)	46 (5.0)
No	88 (98.9)	869 (95.0)

* Data were missing for the following variables: Total monthly household income (1 for cases and 9 for non cases), roof type (1 for non cases), smoking status (4 for cases and 45 for non-cases) and duration of smoking (2 for ex smokers).

Results regarding the association of arsenic concentration and type 2 diabetes are given in Table 3. An increased risk of T2D was observed for arsenic exposure over 50 μg/L with those in the highest quartile category having almost double the risk of type 2 diabetes (OR=1.9 ; 95% CI 1.1-3.5). The odds ratios showed a dose–response pattern with a significant increasing trend in relation with increasing exposure category (p <0.001).We also performed sensitivity analysis excluding participants with lesser duration of exposure (<2years and <5 years) and compared them with participants who were exposed for ≥10 years and did not find any difference to that of a comparison between <10 years and ≥10 years exposure groups.

**Table 3:** Association of arsenic concentration with type 2 diabetes

	Type 2 Diabetes		
	No. of cases	OR*	95% CI
Arsenic concentration (μg/L)			
≤50^†^	33	1.0	-
>50	56	2.1	1.3 – 3.2
Lowest quartile: < 22^†^	19	1.0	-
2^nd^ quartile: 23 - 32	14	1.1	0.5 – 2.3
3^rd^ quartile: 33 – 261	26	1.7	0.5 – 3.2
Highest quartile: ≥262	30	1.9	1.1 – 3.5
*P* for trend^‡^ <0.01			

* Adjusted for participant’s age, sex, education, BMI, and family history of diabetes.

^†^ Reference category.

^‡^ Cuzick's nonparametric test for trend across ordered groups; all four groups were used in trend test.

Therefore, the cumulative effects of arsenic concentration and duration of exposure on T2D are presented in Table 4. The odds ratio estimates show a graded, dose–response pattern as arsenic increases and time exposed increases (p < 0.001). The reference group is those who had concentrations not exceeding 50μg/L for less than 10 years.

**Table 4:** Association of arsenic concentration and duration of exposure with type 2 Diabetes

Duration of arsenic exposure (years)	Type 2 Diabetes			
	Arsenic concentration (μg/L)	No. of cases	OR*	95% CI
	< 50^†^	33^§^	1	-
<10	>50 – 250	10	2.05	0.8 – 5.36
	>250	06	0.68	0.18 – 2.59
≥10	>50 – 250	16	2.11	1.08 – 4.15
	>250	24	2.35	1.28 – 4.32

* Adjusted for participant’s age, sex, bmi and family history of diabetes.

^†^ Reference category.

^§^ In <10 years and ≥10 years exposure groups with ≤50 μg/L arsenic concentration the number of cases are 19 and 14, respectively.

## Discussion

We assessed the risk of T2D in people consuming arsenic contaminated drinking water in Bangladesh. The findings suggest an association between chronic arsenic exposure through drinking water and T2D. Risks are generally higher with longer duration of arsenic exposure. The risk of T2D is highest among those who were exposed to the highest concentration of arsenic (>250μg/L) for more than 10 years.

More females than males participated in this study. This is because data was collected during the morning when many of the males were out of the home for their daily work. The overall prevalence of T2D is higher in this study than that reported previously in rural Bangladesh [25]. The prevalence of T2D is also higher in the low arsenic area of Kaligonj relative to rural areas of Bangladesh. This may be due to increased risk of T2D even at the lower concentration of inorganic arsenic in drinking water [15].

Studies in Taiwan, Bangladesh, and Mexico have all shown an association between high levels of inorganic arsenic in drinking water and increased risk of T2D [5,6,26-28]. These studies are limited by the use of ecologic measures of arsenic in drinking water rather than individual measurements of arsenic exposure through drinking water. Data from the studies in Taiwan and Bangladesh were pooled earlier in a meta-analysis [9] that examined extreme arsenic exposure categories (village drinking water levels or living in a high-vs-low-arsenic area); the pooled relative risk for diabetes associated with high versus low arsenic areas was 2.52 (95% CI: 1.69 – 3.75).

Only a limited number of studies have examined the association of low or moderate exposure to inorganic arsenic with diabetes risk [29,30]. An early ecological study in Utah, USA found no association between arsenic levels in drinking water and diabetes mortality after controlling for age and sex [30]. A cross-sectional study in Wisconsin, USA that examined the association between arsenic in drinking water and nine self reported chronic diseases found that compared to those with an arsenic exposure <2μg/L, those exposed to 2μg/L -10μg/L and >10μg/L of arsenic in drinking water had an adjusted odds ratio of 1.35 (95% CI, 0.78-2.33) and 1.02 (95% CI, 0.49-2.15), respectively [29]. Wang et al., in a previous study found that hair arsenic levels were associated with elevated plasma glucose levels and with the prevalence of the metabolic syndrome in 660 participants exposed to relatively low arsenic levels in drinking water in Taiwan [31]. Chen JW et al in another study from Taiwan reported a two-fold increased risk of T2D among subjects with total urinary As (U-As) >75 μg/g creatinine as compared with individuals whose U-As was <35 μg/g creatinine after adjustment for potential confounders [12]. However, Chen Y et al observed no association between chronic arsenic exposure and diabetes mellitus in Bangladesh and they reported a prevalence of diabetes of 2.1%, low BMI (<20kg/m2) in 49% and exposure to <300μg/L of arsenic in 90% of the participants [14]. On the other hand, our study reports diabetes in 9%, low BMI (<20kg/m^2^) in 55% and exposure to <300μg/L of arsenic in 77% of the participants. The low prevalence of diabetes among arsenic exposed people in the Chen Y et al study might be due to the use of glycated hemoglobin (HbA_1_C) and glucosuria to identify diabetic individuals, rather than fasting blood glucose and 2-hour blood glucose that were used to identify diabetics in another study [28]. Inaccuracies in BMI determination may have contributed to an underestimation of association with T2D risk in our study. Despite the participants in this study might not be representative to the general population; however the participants staying at home were more appropriate for the study, as their exposure to arsenic concentration was determined from the well water they had been consuming.

The mechanism by which arsenic causes T2D is still largely undefined.

Animal model experimentation suggests that arsenic impairs pancreatic β cell function, particularly affecting insulin synthesis and secretion [32,33]. The molecular mechanism for this effect may be via adverse effects on insulin signal transduction and the inhibition of gene transcription factors [34-36]. A few non-specific mechanisms such as oxidative stress, inflammation or apoptosis may also increase the risk of developing diabetes [32].

Important strengths of this study include the use of a large population sample, the direct measurement of arsenic in the subjects’ drinking water, and the adjustment for relevant diabetes risk factors such as age, sex, education, body mass index (BMI) and family history of diabetes. While the public health and research implications of this study are important, some caveats must also be considered. One limitation of this study is that biomarkers of arsenic exposure were not measured. Also, we excluded people with chronic medical conditions which may be related to arsenic exposure, although this would tend to bias towards the null and therefore indicates robustness of our results.

Other limitations include the use of a glucometer to measure fasting glucose as this may give inaccurate results. Validity of this glucometer has not been tested earlier. Therefore, there may be misclassification of T2D. Use of field screening tests to diagnose T2D in relation to explore association with chronic arsenic exposure has been a concern. A few previous studies reported an association between T2D and chronic arsenic exposure using field screening tests such as glucometer for blood, and glucometric strips for glucosuria [28,37]. Laboratory facilities are still rare or unavailable in rural Bangladesh and portable blood glucose devices are suggested for screening rather than diagnosis for diabetes mellitus [38]. Nevertheless, interviewers were unaware of the arsenic concentration levels of the subjects’ usual drinking water. Therefore, this misclassification is likely to be non-differential, and hence may underestimate the risk. It would also have been desirable to have directly measured individual exposure data over time, as the available water samples reflected only a particular point in time and not the historical exposure. In the absence of any reliable information on past exposure, it was essential to assume that arsenic concentrations from the tube wells had been relatively constant over time. The historical consistency of arsenic concentration is of particular concern with shallow ground water, which might be subject to greater fluctuation than water from a deeper well. However, we assume that any fluctuation of arsenic concentrations is likely to affect the study participants equally. Duration of tube well water use was also obtained from the participants. In the absence of any good documentation of water use, we had to rely on the individual’s recall. The study is cross-sectional and temporality between drinking water arsenic and development of T2D cannot be ensured. Prospective epidemiologic studies in populations exposed to a wide range of inorganic arsenic are needed to confirm this association.

Also many other factors need to be considered in any population studies as diabetes risk increases with age, obesity and individual’s physical activity [39]. Another limitation in this study is that we did not adjust for physical activity. However, we adjusted for obesity in the final model. Furthermore, obesity and physical activity would not be an issue in the rural population in Bangladesh as obesity is much lower [40] and physical activity may be higher in this context.

## Conclusions

Our result adds to the growing body of work indicating an association between chronic arsenic exposure and T2D and provides a graded, dose–response relationship. Such an effect could yield insights into the pathophysiology of diabetes and indicate potential future preventive and therapeutic targets in diabetic patients and warrants future larger prospective studies.

## Abbreviations

CCEB, Centre for Clinical Epidemiology and Biostatistics; CHAD, Centre for Health and Development; ICMH, Institute of Child and Mother Health; T2D, Type 2 Diabetes; WHO, World Health Organization; BMI, Body Mass Index; CI, Confidence Interval; OR, Odds Ratio; μg/L, Microgram per liter; BMRC, Bangladesh Medical Research Council; BAMWSP, Bangladesh Arsenic Mitigation Water Supply Project; FIHG-AAS, Flow injection-hydride generation atomic absorption spectrometry; ml, Millilitre; HCl, Hydrochloric Acid; KI, Potassium Iodide; FBG, Fasting blood glucose; TX, Texas; USA, United States of America; Kg/m2, Kilogram per square meter; HbA1C, Glycosylated haemoglobin A1C or a type of haemoglobin that increases in diabetes; HREC, Human Research Ethics Committee.

## Competing interests

We declare that we have no conflict of interests.

## Authors’ contribution

MRI contributed to the conception of the project, proposal writing, data analysis and manuscript writing. IK contributed to conception of the project, proposal writing, data collection and review of the manuscript. SMNH contributed to project supervision and data collection. MM contributed to data analysis and manuscript writing. CD contributed to data analysis and manuscript writing. JA contributed to data analysis and manuscript writing. RP contributed to conception of the project and manuscript writing. MS contributed to project supervision, data collection and manuscript writing. SA contributed to project supervision and manuscript writing. AHM contributed to the conception of the project, proposal writing, data analysis and manuscript writing. All authors read and approved the final manuscript.

## References

[B1] IARC IARC monographs on the evaluation of the carcinogenic risk of chemicals to humans: some metals and metallic compounds 1980 23 39 141 6933135

[B2] NRC Arsenic in drinking water 1999 National research council, Washington

[B3] Rahman M Wingren G Axelson O Diabetes mellitus among Swedish art glass workers–an effect of arsenic exposure? Scand J Work Environ Health 1996 22 2 146 149 10.5271/sjweh.123 8738894

[B4] Rahman M Axelson O Diabetes mellitus and arsenic exposure: a second look at case–control data from a Swedish copper smelter Occup Environ Med 1995 52 11 773 774 10.1136/oem.52.11.773 8535499 PMC1128360

[B5] Rahman M Tondel M Ahmad SA Axelson O Diabetes mellitus associated with arsenic exposure in Bangladesh Am J Epidemiol 1998 148 2 198 203 10.1093/oxfordjournals.aje.a009624 9676702

[B6] Lai MS Hsueh YM Chen CJ Shyu MP Chen SY Kuo TL Wu MM Tai TY Ingested inorganic arsenic and prevalence of diabetes mellitus Am J Epidemiol 1994 139 5 484 492 8154472 10.1093/oxfordjournals.aje.a117031

[B7] Kahn SE Prigeon RL McCulloch DK Boyko EJ Bergman RN Schwartz MW Neifing JL Ward WK Beard JC Palmer JP Quantification of the relationship between insulin sensitivity and beta-cell function in human subjects Evidence for a hyperbolic function. Diabetes 1993 42 11 1663 1672 10.2337/diab.42.11.1663 8405710

[B8] Kahn SE The relative contributions of insulin resistance and beta-cell dysfunction to the pathophysiology of Type 2 diabetes Diabetologia 2003 46 1 3 19 12637977 10.1007/s00125-002-1009-0

[B9] Navas-Acien A Silbergeld EK Streeter RA Clark JM Burke TA Guallar E Arsenic exposure and type 2 diabetes: a systematic review of the experimental and epidemiological evidence Environ Health Perspect 2006 114 5 641 648 16675414 10.1289/ehp.8551 PMC1459913

[B10] Campbell RK Type 2 diabetes: Where we are today: An overview of disease burden, current treatments, and treatment strategies J Am Pharmacists Assoc 2009 49 S3 S9 10.1331/JAPhA.2009.09077 19801365

[B11] Longnecker MP Daniels JL Environmental contaminants as etiologic factors for diabetes Environ Health Perspect 2001 109 Suppl 6 871 876 11744505 10.1289/ehp.01109s6871 PMC1240622

[B12] Chen JW Chen HY Li WF Liou SH Chen CJ Wu JH 2011 The association between total urinary arsenic concentration and renal dysfunction in a community-based population from central Taiwan. Chemosphere, Wang SL 10.1016/j.chemosphere.2011.02.091 21458841

[B13] Lamm SH Engel A Feinleib M Arsenic exposure and diabetes mellitus risk J Occup Environ Med 2006 48 10 1001 1003 author reply 1003–4 10.1097/01.jom.0000237359.75757.25 17033498

[B14] Chen Y Ahsan H Slavkovich V Peltier GF Gluskin RT Parvez F Liu X Graziano JH No association between arsenic exposure from drinking water and diabetes mellitus: a cross-sectional study in Bangladesh Environ Health Perspect 2010 118 9 1299 1305 10.1289/ehp.0901559 20813654 PMC2944093

[B15] Navas-Acien A Silbergeld EK Pastor-Barriuso R Guallar E Arsenic exposure and prevalence of type 2 diabetes in US adults Jama 2008 300 7 814 822 10.1001/jama.300.7.814 18714061

[B16] Zierold KM Knobeloch L Anderson H Prevalence of chronic diseases in adults exposed to arsenic contaminated drinking water Am J Public Health 1994 94 1936 1937 15514231 10.2105/ajph.94.11.1936 PMC1448563

[B17] Paul DS Harmon AW Devesa V Thomas DJ Styblo M Molecular mechanisms of the diabetogenic effects of arsenic: inhibition of insulin signaling by arsenite and methylarsonous acid Environ Health Perspect 2007 115 5 734 742 10.1289/ehp.9867 17520061 PMC1867998

[B18] Kinniburgh DG Smedley PLE Arsenic Contamiantion of Groundwater in Bangladesh. British Geological Survey, Department for International Development, Ministry of Local Government, Rural Developments and Co-operatives 2001 Department of Public Health Engineering,Government of the People's republic of Bangladesh,

[B19] Jelliffe DB The assessment of the nutritional status of the community (with special reference to field surveys in developing regions of the world) Monogr Ser World Health Organ 1966 53 3 271 4960818

[B20] Eaton AD, Clesceri LS, Greenberg AE Standard Methods for the Examination of Water and Wastewater 19 American Public Health Association, American Water Works Association, Water Environment Federation, Washington

[B21] Shetty PS Body mass index, a measurement of chronic energy deficiency in adults 1994 Rome, FAO 7925867

[B22] World Health Organisation Definition and diagnosis of diabetes mellitus and intermediate hyperglycemia.:Report 2006,

[B23] Smith AH Lingas EO Rahman M Contamination of drinking-water by arsenic in Bangladesh: a public health emergency Bull World Health Organ 2000 78 9 1093 1103 11019458 PMC2560840

[B24] Milton AH Smith W Rahman B Hasan Z Kulsum U Dear K Rakibuddin M Ali A Chronic arsenic exposure and adverse pregnancy outcomes in bangladesh Epidemiology 2005 16 1 82 86 10.1097/01.ede.0000147105.94041.e6 15613949

[B25] Hussain A Rahim MA Azad Khan AK Ali SM Vaaler S Type 2 diabetes in rural and urban population: diverse prevalence and associated risk factors in Bangladesh Diabet Med 2005 22 7 931 936 10.1111/j.1464-5491.2005.01558.x 15975110

[B26] Tseng CH Tai TY Chong CK Tseng CP Lai MS Lin BJ Chiou HY Hsueh YM Hsu KH Chen CJ Long-term arsenic exposure and incidence of non-insulin-dependent diabetes mellitus: a cohort study in arseniasis-hyperendemic villages in Taiwan Environ Health Perspect 2000 108 9 847 851 10.1289/ehp.00108847 11017889 PMC2556925

[B27] Wang SL Chiou JM Chen CJ Tseng CH Chou WL Wang CC Wu TN Chang LW Prevalence of non-insulin-dependent diabetes mellitus and related vascular diseases in southwestern arseniasis-endemic and nonendemic areas in Taiwan Environ Health Perspect 2003 111 2 155 159 12573898 10.1289/ehp.5457 PMC1241343

[B28] Del Razo LM García-Vargas GG Valenzuela OL Castellanos EH Sánchez-Peña LC Currier JM Drobná Z Loomis D Stýblo M Exposure to arsenic in drinking water is associated with increased prevalence of diabetes: A cross sectional study in the Zimpan and Lagunera regions in Mexico Environ Heal 2011 10 73 10.1186/1476-069X-10-73 PMC3169452 21864395

[B29] Zierold KM Knobeloch L Anderson H Prevalence of chronic diseases in adults exposed to arsenic-contaminated drinking water Am J Public Health 2004 94 11 1936 1937 10.2105/AJPH.94.11.1936 15514231 PMC1448563

[B30] Lewis DR Southwick JW Ouellet-Hellstrom R Rench J Calderon RL Drinking water arsenic in Utah: A cohort mortality study Environ Health Perspect 1999 107 5 359 365 10.1289/ehp.99107359 10210691 PMC1566417

[B31] Wang SL Chang FH Liou SH Wang HJ Li WF Hsieh DP Inorganic arsenic exposure and its relation to metabolic syndrome in an industrial area of Taiwan Environ Int 2007 33 6 805 811 10.1016/j.envint.2007.03.004 17481731

[B32] Izquierdo-Vega JA Soto CA Sanchez-Peña LC De Vizcaya-Ruiz A Del Razo LM Diabetogenic effects and pancreatic oxidative damage in rats subchronically exposed to arsenite Toxicol Lett 2006 160 2 135 142 10.1016/j.toxlet.2005.06.018 16111841

[B33] Díaz-Villaseñor A Sánchez-Soto MC Cebrián ME Ostrosky-Wegman P Hiriart M Sodium arsenite impairs insulin secretion and transcription in pancreatic beta-cells Toxicol Appl Pharmacol 2006 214 1 30 34 10.1016/j.taap.2005.11.015 16413591

[B34] Macfarlane WM McKinnon CM Felton-Edkins ZA Cragg H James RF Docherty K Glucose stimulates translocation of the homeodomain transcription factor PDX1 from the cytoplasm to the nucleus in pancreatic beta-cells J Biol Chem 1999 274 2 1011 1016 10.1074/jbc.274.2.1011 9873045

[B35] Macfarlane WM Smith SB James RF Clifton AD Doza YN Cohen P Docherty K The p38/reactivating kinase mitogen-activated protein kinase cascade mediates the activation of the transcription factor insulin upstream factor 1 and insulin gene transcription by high glucose in pancreatic beta-cells J Biol Chem 1997 272 33 20936 20944 10.1074/jbc.272.33.20936 9252422

[B36] Elrick LJ Docherty K Phosphorylation-dependent nucleocytoplasmic shuttling of pancreatic duodenal homeobox-1 Diabetes 2001 50 10 2244 2252 10.2337/diabetes.50.10.2244 11574405

[B37] Rahman M Tondel M Chowdhury IA Axelson O Relations between exposure to arsenic, skin lesions, and glucosuria Occup Env Med 1999 56 4 277 281 10.1136/oem.56.4.277 10450246 PMC1757723

[B38] Houweling ST Kleefstra N van Ballegooie E Miedema K Rischen R Heeg JE Diagnosis of diabetes mellitus:Limited use for portable blood-glucose measuring devices Ned Tijdschr Geneeskd 2005 149 13 694 697 15819134

[B39] American Diabetes Association Screening for Diabetes Diabetes Care 2002 25 1 S21 S24

[B40] Hussain A Vaaler S Sayeed MA Mahtab H Ali SM Khan AK Type 2 diabetes and impaired fasting blood glucose in rural Bangladesh: a population-based study Eur J Public Health 2007 17 3 291 296 10.1093/eurpub/ckl235 17008328

